# Pathway Based Analysis of Genes and Interactions Influencing Porcine Testis Samples from Boars with Divergent Androstenone Content in Back Fat

**DOI:** 10.1371/journal.pone.0091077

**Published:** 2014-03-10

**Authors:** Sudeep Sahadevan, Asep Gunawan, Ernst Tholen, Christine Große-Brinkhaus, Dawit Tesfaye, Karl Schellander, Martin Hofmann-Apitius, Mehmet Ulas Cinar, Muhammad Jasim Uddin

**Affiliations:** 1 Institute of Animal Science, University of Bonn, Bonn, Germany; 2 Fraunhofer Institute for Algorithms and Scientific Computing (SCAI), Schloss Birlinghoven, Sankt Augustin, Germany; 3 Department of Animal Production and Technology, Faculty of Animal Science, Bogor Agricultural University, Bogor, Indonesia; 4 Bonn-Aachen International Center for Information Technology (B-IT), Bonn, Germany; 5 Department of Animal Science, Faculty of Agriculture, Erciyes University, Kayseri, Turkey; University of Jaén, Spain

## Abstract

One of the primary factors contributing to boar taint is the level of androstenone in porcine adipose tissues. A majority of the studies performed to identify candidate biomarkers for the synthesis of androstenone in testis tissues follow a reductionist approach, identifying and studying the effect of biomarkers individually. Although these studies provide detailed information on individual biomarkers, a global picture of changes in metabolic pathways that lead to the difference in androstenone synthesis is still missing. The aim of this work was to identify major pathways and interactions influencing steroid hormone synthesis and androstenone biosynthesis using an integrative approach to provide a bird’s eye view of the factors causing difference in steroidogenesis and androstenone biosynthesis. For this purpose, we followed an analysis procedure merging together gene expression data from boars with divergent levels of androstenone and pathway mapping and interaction network retrieved from KEGG database. The interaction networks were weighted with Pearson correlation coefficients calculated from gene expression data and significant interactions and enriched pathways were identified based on these networks. The results show that 1,023 interactions were significant for high and low androstenone animals and that a total of 92 pathways were enriched for significant interactions. Although published articles show that a number of these enriched pathways were activated as a result of downstream signaling of steroid hormones, we speculate that the significant interactions in pathways such as glutathione metabolism, sphingolipid metabolism, fatty acid metabolism and significant interactions in cAMP-PKA/PKC signaling might be the key factors determining the difference in steroidogenesis and androstenone biosynthesis between boars with divergent androstenone levels in our study. The results and assumptions presented in this study are from an in-silico analysis done at the gene expression level and further laboratory experiments at genomic, proteomic or metabolomic level are necessary to validate these findings.

## Introduction

Androgens, the primary hormones secreted by testis control and regulate the development of male accessory reproductive organs and secondary sexual characteristics. In porcine genomics, the special importance given to studying the synthesis and degradation of androgens is mainly due to an androgenic pheromone called androstenone. The accumulation of androstenone in boar adipose tissues is one of the major factors contributing to boar taint [1]. Boar taint is described as an off odor or off taste of meat derived from non castrated male pigs. At present, in many countries, surgical castration of boars is the primary method to reduce boar taint in pork meat [2]. Since piglet castration without anesthesia is going to be banned in the European Community by 2018 due to animal welfare reasons [3], there is an immediate need to develop non surgical methods to reduce boar taint mainly by regulating the synthesis of androstenone. The two proposed non surgical methods to reduce boar taint are: (i) the use of chemicals or drugs to reduce boar taint [4] and (ii) breeding for favorable characteristics to reduce boar taint [5]. In this regard, it should be noted that the European Food Safety Authority (EFSA) has already expressed concerns over consumer perception of meats from animals treated with chemicals and drugs to reduce boar taint [6]. Consumer perception issues over the use of chemicals and drugs for boar taint reduction leaves breeding as a more sustainable and accepted method to adopt for reducing boar taint.

In order to select favorable biomarkers for breeding pigs with low androstenone levels and hence reduce boar taint, it is crucial to understand the genetic machinery behind the synthesis of androstenone. Androstenone is synthesized in testes and metabolized in liver. Although studying genes, interactions and pathways in both testes and liver is essential to understand the entire androstenone metabolic processes, in this study the major focus points are the factors that could contribute to androstenone synthesis in testis tissues. The synthesis of androstenone from pregnenolone in testes is mainly catalyzed by the enzymes cytochrome P450C17 (CYP17A1) and cytochrome B5 (CYB5) along with other reductases. The enzyme 5α reductases (ST5AR) catalyze the final step in the synthesis of androstenone [7]. At this point, it should be taken into account that although a number of enzymes catalyzing various steps in androstenone synthesis have been identified, the entire metabolic processes involved in the synthesis of androstenone has not been understood completely. Nevertheless, several studies have been performed to identify candidate biomarkers related to the synthesis of androstenone in testes. A study focusing on genetic correlations of backfat with direct and associative effects for androstenone has found that direct effects had a genetic correlation of 0.14±0.08 and associative effects had a genetic correlation of −0.25±0.18 [8]. High throughput microarray studies have been conducted to study the difference in gene expression patterns between testis samples from boars with divergent androstenone levels [9], [10]. Additionally, several QTL (Quantitative trait loci) studies and GWAS (genome wide association studies) have also been done to identify candidate QTL regions and polymorphisms responsible for varying levels of androstenone [11]–[16]. An in-house study using data from RNA-seq technology has also been performed recently to identify candidate biomarkers for varying levels of androstenone in porcine testes and liver samples [17]. All these studies have been successful in identifying several candidate QTL regions, genes and polymorphisms as potential candidate biomarkers to pursue further detailed investigations.

A general trend among these aforementioned studies is that the candidate biomarkers identified in these studies are mainly analyzed and explained individually using a reductionist approach. Although individual analysis of candidate biomarkers using a reductionist approach helps to study their functions in detail, a phenotype or a disease is seldom the consequence of a change in a single effector gene or gene product, but rather the result of a multitude of changes in a complex interaction network [18]. From this point of view, integrative approaches merging different data sources with gene expression profiles would be more suited to gain a better understanding of a complex trait such as androstenone. In human development and medicine, integrative analysis approaches merging gene expression profiles with pathway data or interaction network has been shown to be a powerful approach to understand the disease. The usual end result of such methods are diagnostic pathways or disease subnetworks, which are demonstrated to enhance the prediction accuracy of disease states and to be more reproducible than single genes [19]. In this work, we have followed an integrative analysis procedure by merging together interaction network and pathway information from KEGG pathway database along with gene expression data. A current limitation of this approach in terms of studying androstenone metabolism is that none of the major pathway databases contain data on metabolic reaction steps or gene interactions involved in androstenone biosynthesis. As a work around to this limitation, we have treated androstenone biosynthesis as an offshoot of steroid hormone (testosterone) synthesis pathway in testis under the assumption that the pathways and interaction events that affect steroid hormone biosynthesis could also affect androstenone biosynthesis. The major aim of this work was to identify and study the major metabolic pathways and interactions involved in the maintenance and regulation of steroidogenesis and androstenone biosynthesis using gene expression data from porcine testis samples with divergent levels of androstenone measurement through an integrative analysis approach.

## Materials and Methods

### Materials

#### Expression data

The expression dataset used in this study is from a previous in-house RNA seq experiment conducted in order to understand the genetic mechanism behind androstenone metabolism [17]. For the purposes of this work, we used only the ten testis samples from the original study. In the original study, these ten testis samples were selected from a pool of 100 boars. In this pool of animals, boars with a fat androsteone level of 0.5 µg/g or less were defined as low androstenone (LA) animals and boars with a fat androstenone concentration of 1.00 µg/g or more were defined as high androstenone (HA) animals. From this population, 5 animals with extreme high and low levels of androstenone were selected as sample LA and HA population. The average androstenone measurement of LA sample animals was 0.24±0.06 µg/g and the average androstenone measurement of HA sample animals was 2.48±0.56 µg/g. Among these 10 animals, two sets of 3 animals each: 1 LA and 2 HA animals in the first set and 2 LA and 1 HA animals in the second set were half siblings. Additional details of sample collection, library preparation and sequencing are available in [17].

#### Pathway and network data

We retrieved pathway and interaction network data from KEGG database (Release 60.0). This interaction network was comprised of enzyme - enzyme (reaction steps) and protein - protein interactions mapped to the corresponding porcine gene identifiers and annotated with KEGG pathway names and identifiers in which the interactions occur. The interaction network consisted of 23,198 edges (interactions) between 3,510 nodes (genes) mapped to 197 pathways.

### Methods

#### Expression data quality control, mapping and normalization

The first step in expression data analysis was a quality control and filtering step. In this step, PCR primers and bad quality sequences (Phred score <20) identified in the raw reads using FASTQC quality control application [20] were trimmed off. The selection of threshold cut-off (Phred score >20) was arbitrary and yet this cut-off threshold ensured that only the reads with a base quality score of 99% or more were retained for further analysis. The filtered raw reads were mapped to latest *Sus scrofa* genome build, Sscrofa10.2 from NCBI using a “splice aware” mapping algorithm TopHat [21] to generate individual genome mapping files for each sample. The expression set (expression matrix) was created by calculating read counts (expression values) for each gene from these genome mapping files using BEDTools [22]. It has been shown that the read count expression data set generated from an RNA-seq experiment follows a negative binomial distribution [23], but the classical linear modeling analysis procedures developed for microarray data sets assumes the data to be normally distributed. Although various non parametric procedures (distribution free methods) can be used in this context, we found that the results given by such analysis procedures were statistically non significant, owing to the small sample size of our data set and the limited power of non parametric methods to draw significant conclusions from data sets with small sample sizes. Recently, Law et al. [24] proposed applying normal distribution based microarray like statistical analysis methods to RNA-seq read count data. In order to overcome the limitations of small sample sizes and non parametric methods to an extend and also following the proposed idea in [24] of using normal distribution based microarray like statistical analysis methods to RNA-seq read count data, we normalized and log2 transformed our expression data set using “voom” function implemented in limma R package [25]. Comparison of various normalization and differential expression analysis methods for RNA-seq data have shown that voom normalization combined with limma package is relatively unaffected by outliers and performed well under many conditions [26]. An additional study [27] concluded that modeling RNA-seq gene count data as log normal distribution with appropriate pseudo counts (limma voom modeling) is a reasonable approximation of the data. Mean-variance modeling at the observational level (voom) estimates mean-variance relationship in the read count data and computes weights for each observation based on this relationship [24]. Our expression dataset was generated and normalized based on the above mentioned procedure.

##### *Identification of significant interactions*

Since we intended to identify significant interactions based on information from expression data and pathway interaction network, the very first step after quality control and normalization of expression data set was to trim the expression data set for genes in pathway interaction network. There were a total of 2,871 genes in common between both the transformed expression dataset and the interaction network from KEGG database. The trimmed interaction network had 23,198 edges (interactions) between 2,871 nodes (genes). In the next step, we calculated Pearson Correlation Co-efficient (PCC) of gene expression values for both LA and HA expression sets separately and by using these correlation coefficients as edge weight values, we generated two edge weighted interaction networks, (i) LA network where LA correlation coefficients were used as edge weight values and (ii) HA network where HA correlation coefficients were used as edge weight values. At this step, only those correlations with an edge in the interaction network were considered and the remaining correlation coefficients, interactions and genes were excluded from further analysis. Both LA and HA correlation coefficient weighted interaction networks contained 2,871 nodes (genes) and 15,960 edges (interactions) respectively. We termed the interaction network weighted with correlation coefficients from LA samples as “LA network” and the one weighted with correlation coefficients from HA samples as “HA network”. In order to identify the interactions that are significantly different between both LA and HA networks, the edge weights (correlation coefficients) of both the networks were transformed to z-score using Fisher-r-to-z transformation based on the equation:
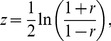
(1)where r is the PCC.

Following the calculation of z-scores for interactions in both networks, the differences between the z-scores were also calculated. For an edge z-score in LA interaction network, the corresponding edge z-score from HA interaction network was retrieved and the difference between the z-scores was calculated as:
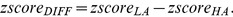
(2)


In order to identify significant z-score_DIFF_ (and there by significant interactions),we followed a two step evaluation criteria based on random sampling and permutation approach [28]. Permutation and resampling based methods for estimating significance thresholds have already been used in high throughput studies [29], [30]. The evaluation criteria used in this study were: (i) zscore_DIFF_ should be significant at a threshold of empirical p-value <0.05 against a set of z-scores randomly generated from the original expression data and (ii) at least one of the correlation coefficients used to calculate zscore_DIFF_ (in equation 2) should be significant at a threshold of empirical p-value <0.05 against a set of correlation coefficients randomly generated from the original expression data. For generating the set of z-scores in evaluation criteria (i) the first step was to generate a random expression matrix by randomly shuffling and reassigning the expression values into two sample groups. By this random shuffling and reassigning, we aimed to break up the original ordering and classification of the expression values and generate two complete random expression matrices and artificially replicate a set of z-scores calculated from a random population. Pearson correlation coefficients, random z-scores and z-score differences were calculated on these random expression matrices following the steps described previously. This entire process was repeated 10,000 times to generate a set of random z-score differences (zscore_RAND_) for each interaction. Significance threshold empirical p-value for each zscore_DIFF_ was calculated as:
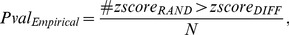
(3)where N = 10,000.

A similar procedure was followed for calculating significance threshold empirical p-value for correlations in evaluation criteria (ii), where empirical p-values were calculated between correlation coefficients from randomly sampled expression data (random population correlation coefficients) and the original correlation coefficients from LA or HA datasets.

Selecting only significant zscore_DIFF_s for further analysis would imply that we were selecting only those gene – gene interactions with a significant difference between LA and HA z-scores when compared to the set of random population z-scores. By adding the additional criterion that at least one of the correlation coefficients used to calculate the zscore_DIFF_ should be significantly different from the set of random population correlation coefficients, we ensured that the selected gene – gene interactions had not only significant zscore_DIFF_s but also at least one significant correlation coefficient when compared to the random population data. We termed these selected interactions as “significant interactions”, since zscore_DIFF_ defined for these interactions (edges) and at least one of the correlation coefficients used to calculate zscore_DIFF_ were significant with respect to random population data. Once the identification of significant interaction was complete, we further classified these significant interactions into 8 interaction types such as: HA positive, HA positive significance, HA negative, HA negative significance, LA positive, LA positive significance, LA negative and LA negative significance. The rules used for classification of these interaction types and edge colors and line styles used in visualization of these interaction types are given in Table 1. These classification rules were mainly used in the visualization step, and all the interaction networks in this work were visualized using Cytoscape [31]. Figure S1 shows a schematic diagram of the entire workflow used in this analysis.

**Table 1:** Interaction edge classification rules.

Correlation type	Correlation coefficient in HA samples	Correlation coefficient in LA samples	Edge color for visualization	Edge line style for visualization
HA positive	positive and significant	negative	red	solid line
HA positive significance	positive and significant	positive	red	dashed line
HA negative	negative and significant	positive	light green	solid line
HA negative significance	negative and significant	positive or negative	light green	dashed line
LA positive	negative	positive and significant	green	solid line
LA positivesignificance	positive	positive and significant	green	dashed line
LA negative	positive	negative and significant	orange	solid line
LA negative significance	positive or negative	negative and significant	orange	dashed line

Set of rules used for the classification of interactions (correlations) and assigning interaction types, edge color and line styles.

Once the identification and classification of significant interactions were completed, we performed a hypergeometric test to identify the pathways over-represented for these significant interactions. The purpose of performing a hypergeometric test here was to test whether there the overlap between the gene interactions to pathway mappings from KEGG database and the interactions identified in the steps above was significant. The hypergeometric test we used is an in-built function (phyper) available in R statistical environment [32] and the probability values generated by the phyper function were converted into p-values (1-probability) and were corrected for multiple testing using Benjamini-Hochberg procedure (BH-correction). All the pathways with a p-adjusted value significance threshold of p-adj <0.05 from hypergeometric tests were considered as significantly enriched pathways.

### **Results and Discussion**

**Figure 1:**
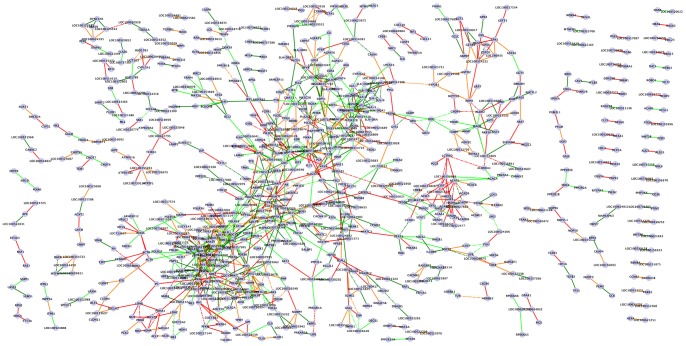
Network visualization of significant interactions identified. Legend: nodes – genes, edges – interactions with significant z-scores. Edge legend: Red solid edges: interactions positive and significant in HA samples, negative in LA samples. Red dashed edges: interactions positive and significant in HA samples, positive in LA samples. Orange solid edges: interactions positive in HA samples, negative and significant in LA samples. Orange dashed edges: interactions negative in HA samples, negative and significant in LA samples. Dark green solid edges: interactions positive and significant in LA samples, negative in HA samples. Dark green dashed edges: interactions positive and significant in LA samples, positive in HA samples. Light green solid edges: interactions positive in LA samples, negative and significant in HA samples. Light green dashed edges: interactions negative in LA samples, negative and significant in HA samples.

Results from our analysis show that 1,023 interactions between 826 genes were significant in LA and HA data sets. Network analysis revealed that these 1,023 interactions formed into an interaction network and the largest connected component of this network contained 848 edges (interactions) and 563 nodes (genes) (Figure 1). File S1 (Cytoscape .xgmml file) contains the significant interactions visualized as a network along with additional information such as LA and HA correlation coefficients, raw read counts for each gene, empirical p-value and correlation type for each interaction. Node degree (number of interactions of a gene) calculations done on this network revealed that genes such as LOC100623707 (POLR2G), ADCY9, PDE8B, NUDT2, PDE8B and LOC100620235 (PIK3R1) were some of the highly connected genes in this network. Among the significant interactions in this network, 209 interactions were LA positive, 201 interactions were LA negative, 257 interactions were HA positive and 220 interactions were HA negative (Table 2). Among the genes involved in significant interactions, gene CYP17A1 is discussed as a candidate gene for androstenone biosynthesis in a number of publications [9], [10], [33], [34] due to its role in the conversion of 17 α-Hydroxy progesterone into androstenedione, a preliminary step in the synthesis of androstenone and testosterone [35]. Additionally, the gene LOC100620470 (HSD17B6) is previously reported to be in an androstenone related QTL region [14] and was also involved in significant interactions in this study. 17 β-hydroxysteroid dehydrogenase type 6 enzyme encoded by this gene catalyzes the conversion of testosterone back to androstenedione [36]. The gene SMPD1, involved in significant interactions in this study, was shown to be downregulated in high androstenone Duroc animals, however this result was not confirmed in rcPCR (real competitive PCR) validation [10]. It was shown that the enzyme encoded by SMPD1 cleaves sphingomyelin to ceramide, which in turn inhibits CYP19, a gene catalyzing a number of reactions in the synthesis of cholesterol, steroids and other lipids [37].

**Table 2:** Network statistics table.

	Significant interactions	Significant interactionsin enriched pathways
**Edges (Interactions)**	1,023	848
**Nodes (genes)**	826	563
**LA positive interactions**	209	173
**LA positive significance interactions**	35	31
**LA negative interactions**	201	166
**LA negative significance interactions**	30	24
**HA positive interactions**	257	217
**HA positive significance interactions**	42	39
**HA negative interactions**	220	189
**LA negative significance interactions**	29	26

This table contains basic information on networks generated from significant interactions and significant interactions in enriched pathways.

The major aim behind pathway enrichment analysis was to relate significant interactions to metabolic pathways and to identify the key pathways and interactions that might be relevant for porcine testicular steroidogenesis and androstenone synthesis. Pathway enrichment analysis showed that out of 1,023 significant interactions, 865 interactions between 718 genes were enriched in 92 pathways (Table 3). File S2 (Cytoscape .xgmml file) contains network visualization of significant interactions in enriched pathways and each edge in this network holds attributes containing KEGG pathway identifiers and names of enriched pathways. Among these enriched pathways, the top 5 enriched pathways in terms of the number of interactions were: purine, pyrimidine and glycerophospholipid metabolism pathways, phosphatidylinositol signaling system and Jak-STAT signaling pathway (Table 3). The significant interactions in pathways such as synthesis and degradation of ketone bodies, steroid biosynthesis, oxidative phosphorylation, butanoate metabolism, drug metabolism – other enzymes and RNA transport were found only in HA samples and the interactions in antigen processing and presentation pathway, intestinal immune network for IgA production, autoimmune thyroid disease and allograft rejection pathways were found only in case of LA sample set (Table 3). Although the pathways mentioned here were some of the top enriched pathways in our analysis, literature references [38]–[43] suggest that a number of these pathways were activated by steroid hormones through various signaling pathways and did not influence steroidogenesis. However, some of the enriched pathways of our interest were: steroid hormone biosynthesis pathway, fatty acid metabolism, oxidative phosphorylation, glutathione metabolism and sphingolipid metabolism. These pathways were chosen as pathways of interest since steroid hormone biosynthesis is the major pathway synthesizing testosterone and androstenone and since literature based evidences suggest that metabolites from glutathione metabolism, sphingolipid metabolism and fatty acid metabolism can influence steroid hormone biosynthesis [44]–[46]. Based on these enriched pathways and significant interactions, we have formalized five major assumptions on the synthesis and maintenance of steroidogenesis and androstenone metabolism in our porcine testis samples. These assumptions are discussed below.

**Table 3:** Pathways enriched for significant interactions.

Id	Name	P-adj.value	# total significantinteractions	# significant interactionsin LA samples	# significant interactionsin HA samples
ssc00230	Purine metabolism	0	187	94	93
ssc00240	Pyrimidine metabolism	0	74	37	37
ssc00564	Glycerophospholipid metabolism	0	55	29	26
ssc04070	Phosphatidylinositol signaling system	0.000006	44	24	20
ssc04630	Jak-STAT signaling pathway	0.000357	33	22	11
ssc05200	Pathways in cancer	0.000024	32	23	9
ssc00280	Valine, leucine and isoleucine degradation	0	23	5	18
ssc00071	Fatty acid metabolism	0	23	3	20
ssc04141	Protein processing in endoplasmicreticulum	0	23	10	13
ssc00600	Sphingolipid metabolism	0.000456	21	11	10
ssc04510	Focal adhesion	0.033436	21	12	9
ssc00561	Glycerolipid metabolism	0.000055	21	12	9
ssc04010	MAPK signaling pathway	0.022251	20	7	13
ssc04612	Antigen processing and presentation	0	19	19	0
ssc04370	VEGF signaling pathway	0	19	8	11
ssc00480	Glutathione metabolism	0.000055	19	6	13
ssc05166	HTLV-I infection	0.005501	18	7	11
ssc05416	Viral myocarditis	0	17	16	1
ssc00620	Pyruvate metabolism	0.000001	17	5	12
ssc04514	Cell adhesion molecules (CAMs)	0.000003	17	16	1
ssc04530	Tight junction	0.023193	17	8	9
ssc05320	Autoimmune thyroid disease	0	16	16	0
ssc05330	Allograft rejection	0	16	16	0
ssc04910	Insulin signaling pathway	0.002451	16	6	10
ssc04114	Oocyte meiosis	0.000002	16	5	11
ssc00565	Ether lipid metabolism	0.001179	16	11	5
ssc04662	B cell receptor signaling pathway	0.000022	14	6	8
ssc05152	Tuberculosis	0.00428	14	5	9
ssc04660	T cell receptor signaling pathway	0.002445	13	5	8
ssc00640	Propanoate metabolism	0	13	3	10
ssc00010	Glycolysis/Gluconeogenesis	0.000011	13	2	11
ssc04520	Adherens junction	0.000045	12	2	10
ssc05215	Prostate cancer	0.002451	12	5	7
ssc04650	Natural killer cell mediated cytotoxicity	0.023263	11	3	8
ssc00350	Tyrosine metabolism	0.000006	11	7	4
ssc04914	Progesterone-mediated oocyte maturation	0.000006	11	6	5
ssc04145	Phagosome	0.000104	11	5	6
ssc04360	Axon guidance	0.012443	10	5	5
ssc04720	Long-term potentiation	0.033436	9	1	8
ssc00330	Arginine and proline metabolism	0.002441	9	7	2
ssc03013	RNA transport	0.027555	9	0	9
ssc05221	Acute myeloid leukemia	0.001945	9	4	5
ssc00520	Amino sugar and nucleotide sugarmetabolism	0.004446	9	2	7
ssc00760	Nicotinate and nicotinamide metabolism	0.006482	8	4	4
ssc00140	Steroid hormone biosynthesis	0.022251	8	3	5
ssc00650	Butanoate metabolism	0.000053	8	0	8
ssc00020	Citrate cycle (TCA cycle)	0.002123	8	1	7
ssc05214	Glioma	0.039816	8	4	4
ssc05218	Melanoma	0.018072	8	4	4
ssc04672	Intestinal immune network for IgA production	0.000006	7	7	0
ssc05310	Asthma	0.000002	7	7	0
ssc05323	Rheumatoid arthritis	0.00002	7	7	0
ssc05322	Systemic lupus erythematosus	0.000002	7	7	0
ssc04210	Apoptosis	0.025555	7	2	5
ssc00260	Glycine, serine and threonine metabolism	0.012235	7	2	5
ssc03015	mRNA surveillance pathway	0.006833	7	3	4
ssc05145	Toxoplasmosis	0.014424	7	4	3
ssc00190	Oxidative phosphorylation	0.003872	7	0	7
ssc00670	One carbon pool by folate	0.004358	7	4	3
ssc04012	ErbB signaling pathway	0.019074	7	1	6
ssc04976	Bile secretion	0.016901	7	6	1
ssc05210	Colorectal cancer	0.000576	7	4	3
ssc05212	Pancreatic cancer	0.000926	7	6	1
ssc00360	Phenylalanine metabolism	0.002441	6	3	3
ssc00270	Cysteine and methionine metabolism	0.018955	6	4	2
ssc03008	Ribosome biogenesis in eukaryotes	0.002101	6	1	5
ssc03018	RNA degradation	0.016476	6	2	4
ssc05160	Hepatitis C	0.008178	6	4	2
ssc05220	Chronic myeloid leukemia	0.016476	6	2	4
ssc04150	mTOR signaling pathway	0.004199	6	3	3
ssc00380	Tryptophan metabolism	0.011081	5	3	2
ssc00410	beta-Alanine metabolism	0.029887	5	1	4
ssc05010	Alzheimers disease	0.000887	5	2	3
ssc00250	Alanine, aspartate and glutamate metabolism	0.024271	5	2	3
ssc05223	Non-small cell lung cancer	0.023193	5	1	4
ssc05030	Cocaine addiction	0.033436	4	2	2
ssc00340	Histidine metabolism	0.004757	4	3	1
ssc00072	Synthesis and degradation of ketone bodies	0.000456	4	0	4
ssc00052	Galactose metabolism	0.002123	4	3	1
ssc05222	Small cell lung cancer	0.035287	4	1	3
ssc05211	Renal cell carcinoma	0.027555	4	1	3
ssc04621	NOD-like receptor signaling pathway	0.005501	3	2	1
ssc04622	RIG-I-like receptor signaling pathway	0.018072	3	2	1
ssc05134	Legionellosis	0.006482	3	2	1
ssc00983	Drug metabolism - other enzymes	0.015249	3	0	3
ssc05213	Endometrial cancer	0.047691	3	1	2
ssc05216	Thyroid cancer	0.008873	3	3	0
ssc00563	Glycosylphosphatidylinositol(GPI)-anchorbiosynthesis	0.016476	3	0	3
ssc05412	Arrhythmogenic right ventricularcardiomyopathy (ARVC)	0.002578	2	1	1
ssc00740	Riboflavin metabolism	0.016358	2	2	0
ssc04962	Vasopressin-regulated water reabsorption	0.027043	2	0	2
ssc00100	Steroid biosynthesis	0.047448	2	0	2

### Steroid Hormone Synthesis

**Figure 2:**
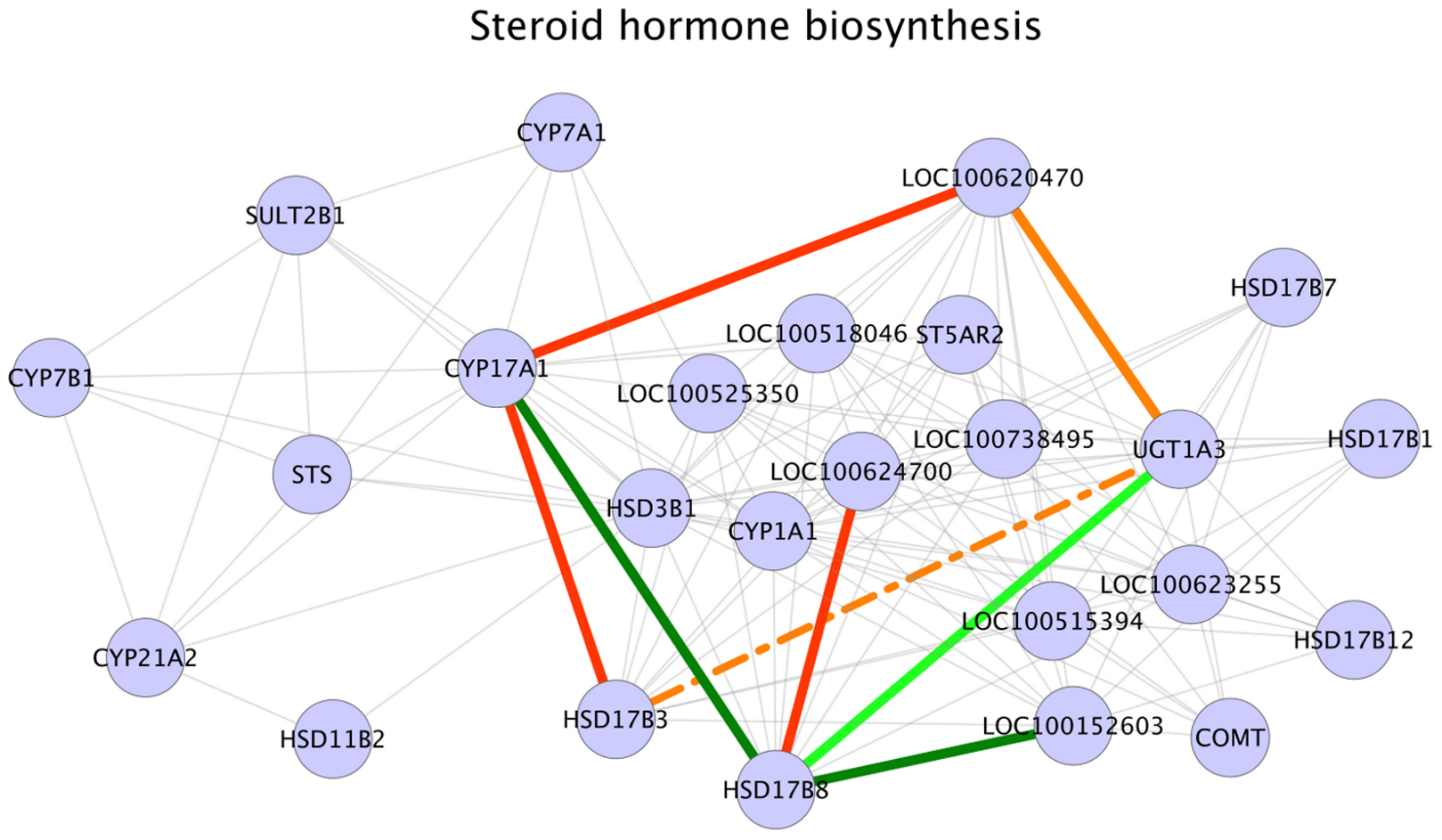
Steroid hormone synthesis pathway. Legend: nodes – genes, edges – interactions with significant z-scores. Edge legend: Grey edges: Non significant interactions, part of KEGG network data. Red solid edges: interactions positive and significant in HA samples, negative in LA samples. Red dashed edges: interactions positive and significant in HA samples, positive in LA samples. Orange solid edges: interactions positive in HA samples, negative and significant in LA samples. Orange dashed edges: interactions negative in HA samples, negative and significant in LA samples. Dark green solid edges: interactions positive and significant in LA samples, negative in HA samples. Dark green dashed edges: interactions positive and significant in LA samples, positive in HA samples. Light green solid edges: interactions positive in LA samples, negative and significant in HA samples. Light green dashed edges: interactions negative in LA samples, negative and significant in HA samples.

As expected, steroid hormone biosynthesis pathway is one of the pathways enriched for significant interactions (Table 3). In this pathway, five significant interactions (correlations) were positive in HA sample set and three significant interactions were positive in LA sample set (Figure 2). One of the interactions positive in HA sample set was the interaction between the genes CYP17A1 and HSD17B3 (Figure 2). The enzyme encoded by CYP17A1 gene converts 17 α-Hydroxy progesterone into androstenedione [35] and the hydroxysteroid dehydrogenase enzyme encoded by HSD17B3 gene catalyzes the conversion of androstenedione to testosterone [47]. Another HA positive interaction in our results was the interaction between the genes CYP17A1 and LOC100620470 (HSD17B6) (Figure 2). In this second interaction involving CY17A1 gene, the interactant was LOC100620470 (HSD17B6). As discussed above, this gene encodes 17 β-hydroxysteroid dehydrogenase type 6 enzyme, which catalyzes the conversion of testosterone back to androstenedione [36]. The third HA positive interaction in steroid hormone biosynthesis pathway was the interaction between the genes LOC100620470 (HSD17B6) and UGT1A3 (Figure 2). The enzyme encoded by UGT1A3 gene, a LOC100620470 (HSD17B6) interaction partner catalyzes the glucuronidation of testosterone to testosterone glucuronide [48]. The fourth HA positive interaction in this pathway was between genes HSD17B8 and LOC100624700 (UGT2C1) (Figure 2). Among these interaction partners, the former codes for the enzyme hydroxysteroid (17-beta) dehydrogenase 8, primarily involved in testosterone inactivation [49] and the latter encodes UDP-glucuronosyltransferase 2C1 enzyme. Although the enzyme UDP-glucuronosyltransferase 2C1 is known to catalyze the conjugation of endogenous compounds, its exact function in relation with hydroxysteroid dehydrogenase enzyme remains unclear. The final positive interaction in HA samples was the interaction between genes HSD17B3 and UGT1A3 (Figure 2). As described above, the enzyme encoded by HSD17B3 converts androstenedione to testosterone and UGT1A3 gene product catalyzes the glucuronidation of testosterone to testosterone glucuronide. The evidences described here could indicate that both testosterone synthesis and degradation steps were active in HA sample set. In case of LA sample set, positive interactions were CYP17A1– HSD17B8 interaction, HSD17B8– UGT1A3 interaction and HSD17B8 - LOC100152603 (UDP-glucuronosyltransferase) interaction (Figure 2). As mentioned above, CYP17A1 codes for an enzyme catalyzing 17α-Hydroxy progesterone to androstenedione conversion and the enzyme hydroxysteroid (17-beta) dehydrogenase 8 encoded by HSD17B8 gene inactivates testosterone. The remaining interaction partners of HSD17B8 gene, UGT1A3 and LOC100152603 (UDP-glucuronosyltransferase) primarily catalyzes the conjugation and removal of various endogenous compounds. It should be noted that in all the three interactions positive in LA sample sets, the gene HSD17B8 was one of the interaction partners and the major function of the protein encoded by this gene is testosterone inactivation. These results and evidences could be an indication that in low androstenone animals, testicular testosterone concentrations were primarily affected by a low amount of synthesis coupled with active testosterone inactivation and degradation steps. A recent study [50] has shown that estimated breeding value of androstenone was positively related to plasma testosterone levels and it was also shown that genetic correlation between androstenone (plasma and fat) and sex steroids were high in pure bred Duroc and Landrace populations [51]. Based on these evidences from published studies and the observation that the enzymes involved in the synthesis of testosterone also catalyzes androstenone synthesis and since both the compounds are derived from pregnenolone [7], we postulate that in HA animals, an active testosterone synthesis could also imply active synthesis of androstenone.

### Glutathione Metabolism

**Figure 3:**
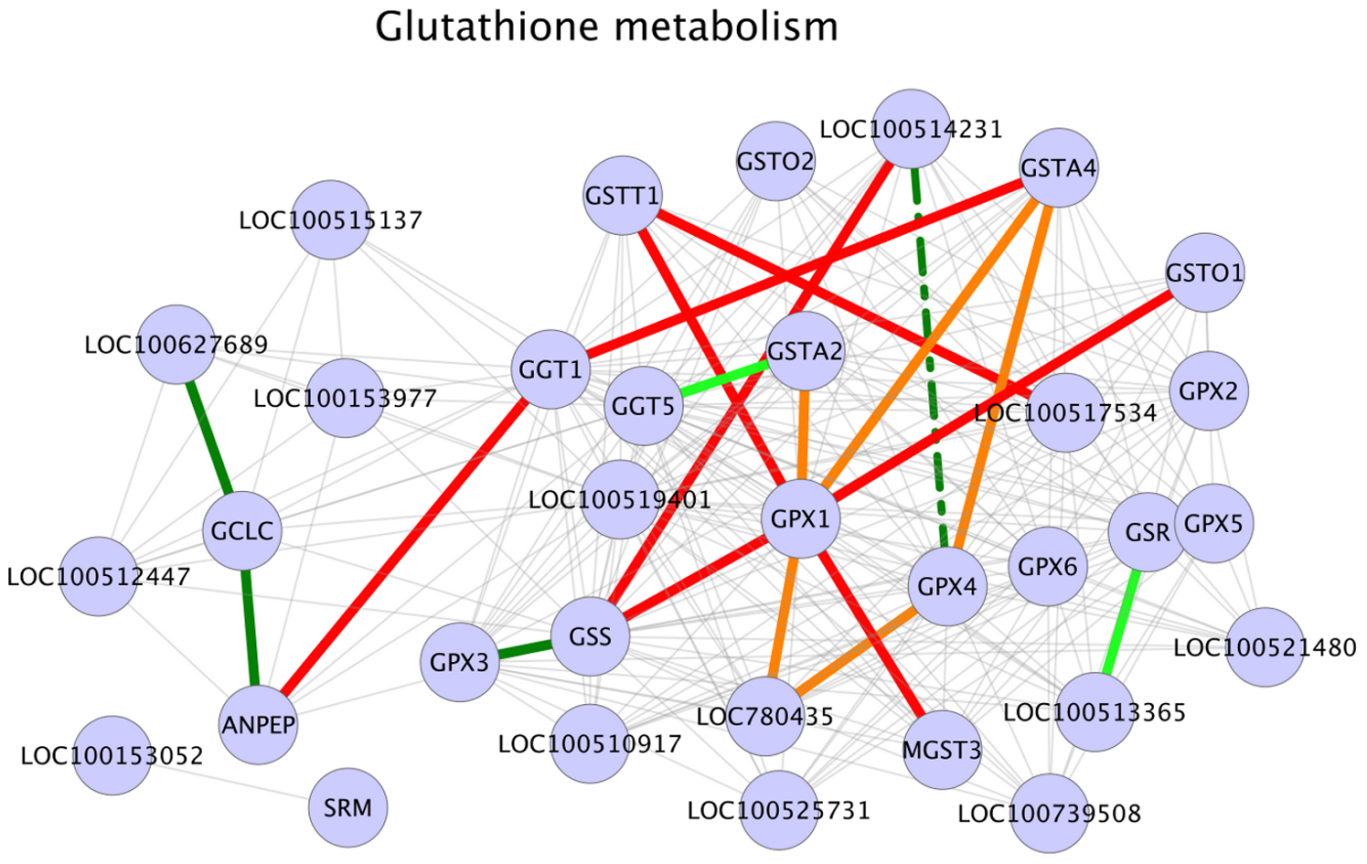
Glutathione metabolism. Legend: nodes – genes, edges – interactions with significant z-scores. Edge legend: Grey edges: Non significant interactions, part of KEGG network data. Red solid edges: interactions positive and significant in HA samples, negative in LA samples. Red dashed edges: interactions positive and significant in HA samples, positive in LA samples. Orange solid edges: interactions positive in HA samples, negative and significant in LA samples. Orange dashed edges: interactions negative in HA samples, negative and significant in LA samples. Dark green solid edges: interactions positive and significant in LA samples, negative in HA samples. Dark green dashed edges: interactions positive and significant in LA samples, positive in HA samples. Light green solid edges: interactions positive in LA samples, negative and significant in HA samples. Light green dashed edges: interactions negative in LA samples, negative and significant in HA samples.

**Figure 4:**
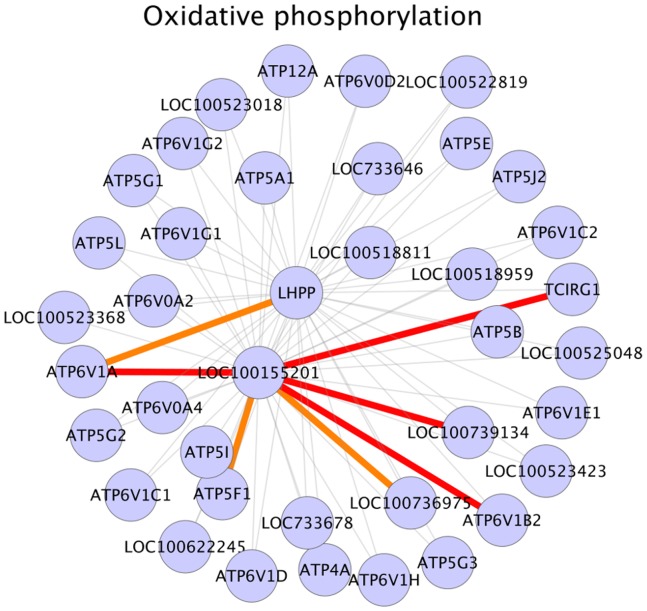
Oxidative phosphorylation. Legend: nodes – genes, edges – interactions with significant z-scores. Edge legend: Grey edges: Non significant interactions, part of KEGG network data. Red solid edges: interactions positive and significant in HA samples, negative in LA samples. Red dashed edges: interactions positive and significant in HA samples, positive in LA samples. Orange solid edges: interactions positive in HA samples, negative and significant in LA samples. Orange dashed edges: interactions negative in HA samples, negative and significant in LA samples. Dark green solid edges: interactions positive and significant in LA samples, negative in HA samples. Dark green dashed edges: interactions positive and significant in LA samples, positive in HA samples. Light green solid edges: interactions positive in LA samples, negative and significant in HA samples. Light green dashed edges: interactions negative in LA samples, negative and significant in HA samples.

Glutathione metabolism was another major metabolic pathway enriched for significant correlations (interactions) in our results (Table 3). Literature evidence suggests that the depletion of intracellular gluathione pool significantly decreases testosterone production [44] and that a decrease in glutathione peroxidase (Gpx) activity affects testosterone synthesis since Gpx activity reduces lipid peroxidation [52]. Additionally, it has also been indicated that alterations in glutathione redox cycle might play significant roles in detoxifying mechanisms in testes [53]. Our analysis identified seven GPX1 gene interactions to be positive in HA sample set (Figure 3). GPX1 gene encodes glutathione peroxidase enzyme, primarily involved in the detoxification of hydrogen peroxide. GSTA2, a GPX1 interaction partner in glutathione metabolism pathway exhibits high activity against lipid peroxidation [54]. GSTA4, another GPX1 interactant metabolizes lipid peroxidation product 4-hydroxynonenal (4-HNE) by conjugating it with glutathione (GSH) [55]. GPX1– GSTA2 interaction (correlation) and GPX1– GSTA4 interaction (correlation) were positive in HA phenotype, possibly indicating that the combined action of enzymes encoded by these genes reduced lipid peroxidase activity in HA samples and thus had a positive effect on testicular steroidogenesis. In this scenario, it should also be taken into account that the majority of reactive oxygen species (ROS), the primary agent in lipid peroxidation is a by-product of mitochondrial oxidative phosphorylation [56]. Our pathway enrichment analysis and further investigations have shown that oxidative phosphorylation pathway was enriched for significant interactions (Table 3) and that a number of interactions (correlations) in oxidative phosphorylation pathway were positive in HA dataset (Table 3, Figure 4). From these results it could be assumed that in HA samples, an active glutathione metabolism pathway might be balancing the negative side effects of an active mitochondrial oxidative phosphorylation, specifically, the peroxidation of lipids triggered by ROS. Interaction evidences also shows the gene GGT1 as an interaction partner for the gene GSTA4 and that the interactions were positive in HA dataset (Figure 3). Conversion of glutathione (GSH) into cysteinyl glycine and γ-glutamate catalyzed by GGT1 gene product is an essential step that helps to maintain cellular levels of glutathione and cysteine and GGT1 deficient male mice have been shown to be infertile [57]. Although KEGG interaction network includes an interaction between GSTA4 and GGT1, at this point we are unable to identify any published evidence supporting this interaction. Based on the evidences stated above, it could be postulated that in HA testis tissues, an active glutathione metabolic pathway resulted in reduced lipid peroxidase activity and thus an increased steroidogenesis and androstenone biosynthesis. In this regard, we propose the genes GPX1 and its interactions partners such as GST family genes GSTA4 and GSTA2 and gene GGT1 in glutathione metabolism as candidate biomarkers to study for their involvement in porcine testicular steroid biosynthesis and androstenone biosynthesis. Among the genes involved in significant interactions in this pathway, the gene GSTO1 is previously reported to be differentially expressed in high androstenone (Duroc) boars [10].

### Sphingolipid Metabolism

**Figure 5:**
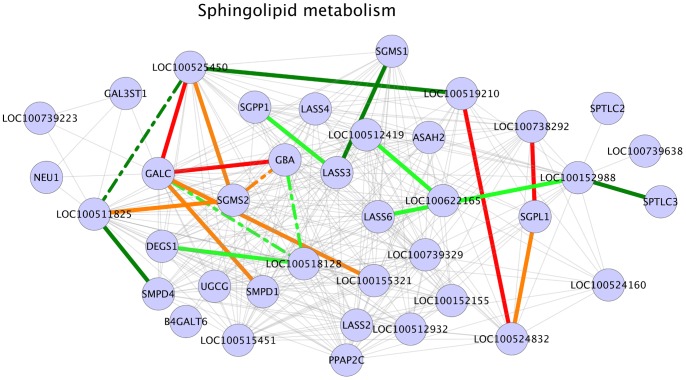
Sphingolipid metabolism. Legend: nodes – genes, edges – interactions with significant z-scores. Edge legend: Grey edges: Non significant interactions, part of KEGG network data. Red solid edges: interactions positive and significant in HA samples, negative in LA samples. Red dashed edges: interactions positive and significant in HA samples, positive in LA samples. Orange solid edges: interactions positive in HA samples, negative and significant in LA samples. Orange dashed edges: interactions negative in HA samples, negative and significant in LA samples. Dark green solid edges: interactions positive and significant in LA samples, negative in HA samples. Dark green dashed edges: interactions positive and significant in LA samples, positive in HA samples. Light green solid edges: interactions positive in LA samples, negative and significant in HA samples. Light green dashed edges: interactions negative in LA samples, negative and significant in HA samples.

Sphingolipids are a class of lipids composed of an aliphatic amino alcohols and a sphingosine (long chain base) backbone. These lipids have been established to play a significant role in steroidogenic pathway by acting as secondary messengers, paracrine, autocrine regulators and nuclear receptors [45]. Literature evidences [58], [59] show that ceramides (Cer, N-acylsphingosine), a major class of sphingolipids can suppress testicular StAR gene expression, testosterone biosynthesis and regulate hCG stimulated steroidogenesis in rat Leydig cells. Studies have also shown that sphingosine-1-phosphate (S1P), an intracellular sphingolipid inhibits germ cell apoptosis in human testis [60] and modulates lutenizing hormone signaling [61]. Sphingomyelin, another sphingolipid is shown to enhance steroid hormone synthesis [62]. It is also suggested that sphingosine (SPH), another sphingolipid class member acts as an antagonist for steroid hormone biosynthesis nuclear receptor SF1 [63]. Sphingolipid metabolism was one of the pathways found to be enriched for significant interactions in our results (Table 3). A total of 10 interactions in this pathway were positive for HA samples (Figure 5). Among these HA positive interactions, gene GALC was involved in 4 interactions (Figure 5). The protein encoded by this gene hydrolyzes the galactose ester double bonds of various sphingolipids including galactoceramide and converts into N-acylsphingosine (ceramide) [64]. The first interaction partner of GALC was the gene SMPD1, which encodes a sphingomyelinase enzyme that converts sphingomyelin to ceramide [64]. GBA gene was the second HA positive GALC interaction partner and the product of this gene hydrolyzes D-glucosyl-N-acylsphingosine to D-glucose and N-acylsphingosine. LOC100155321 (ACER2) was the third GALC interactant in HA positive interactions and the product of this gene catalyzes the hydrolysis of N-acylsphingosine to sphingosine [65]. In case of gene LOC100525450 (CERS1), the final GALC interaction partner in HA positive interactions, it is speculated that the enzyme encoded by this gene is either a ceramide synthase or a modulator. Although ceramide synthases have been shown to catalyze the de novo synthesis of ceramides [66], we were unable to find the function of the gene LOC100525450 (CERS1) or its product in relation to GALC. The results also show that three interactions involving the gene SGMS2 were also positive in HA samples (Figure 5). The enzyme encoded by the gene SGMS2 is involved in the synthesis of sphingomyelin from ceramides [67]. The interaction partners of SGMS2 in HA positive interactions were the genes LOC100525450 (CERS1), GBA and LOC100511825 (UGT8). As mentioned above, the product of the gene LOC100525450 (CERS1) is speculated to be a ceramide synthase or a modulator and the enzyme encoded by the GBA gene hydrolyzes D-glucosyl-N-acylsphingosine to D-glucose and ceramide. The enzyme encoded by LOC100511825 (UGT8) catalyzes the transfer of galactose to ceramide during the synthesis of galactocerebrosides [68]. An additional HA positive interaction in this pathway was the interaction between the genes LOC100738292 (SPHK2) and SGPL1. LOC100738292 (SPHK2) gene product phosphorylates sphingosine to sphingosine-1-phosphate [69]. The enzyme encoded by the gene SGPL1 cleaves sphingoid bases such as sphingosine-1-phosphate into fatty aldehydes and phosphoethanolamine [70]. From these evidences at the gene level, it could be speculated that in HA samples, ceramides were mainly generated by the conversion/hydrolysis of other sphingolipids such as sphingomyelin or D-glucosyl-N-acylsphingosine and that the ceramides generated were converted to galactocerebrosides or to sphingosine and finally into fatty aldehydes and phosphoethanolamine. In our results, a total of 11 interactions in sphingolipid metabolic pathway were positive for LA samples (Figure 5). The gene LOC100152988 (KDSR) was involved in two out of 11 LA positive interactions (Figure 5). One of the interaction partners of LOC100152988 (KDSR) was the gene SPTLC3. The enzyme encoded by SPTLC3 converts palmitoyl-CoA and L-serine into 3-ketodihydrosphingosine, initiating de novo synthesis of sphingolipids [71]. The reductase enzyme encoded by LOC100152988 (KDSR) reduces 3-ketodihydrosphingosine into dihydrosphingosine [72]. The second interaction partner of LOC100152988 (KDSR) was the gene LASS6. LASS6 gene encodes a ceramide synthase enzyme, Ceramide synthase 6 and it is shown that ceramide synthases (CerS) are involved in the acylation of dihydro sphingosine to dihydroceramide, a precursor of ceramide [66]. From these interactions it could be speculated that sphingolipid de novo synthesis was active in case of LA samples. Similar to HA samples, an interaction between a gene coding for an enzyme involved in the synthesis of sphingomyelin and a gene coding for ceramide synthase or modulator was found to be positive in LA animals. This interaction was between the genes LASS3 and SGMS1 (Figure 5). An interaction between the genes LOC100512419 (PPAP2B) and LOC100622165 (ACER1) was also found to be LA positive. LOC100512419 (PPAP2B) hydrolyzes sphingosine-1-phosphate [67] and LOC100622165 (ACER1) hydrolyzes ceramide to sphingosine. Literature based evidences [45], [58]–[63], [73], [74] indicate that elevated amounts of ceramide negatively affects steroid biosynthesis and our evidences at the genomic level suggest active de novo sphingolipid synthesis steps in LA animals. Based on these genomic level evidences, we postulate that elevated concentrations of ceramide in LA animals could be one of the contributing factors to reduced steroid synthesis and possibly reduced androstenone biosynthesis in this phenotype. Although there were several interactions positive in HA animals suggesting the conversion of various sphingolipids to ceramide in these animals, we speculate that ceramide levels in these animals were maintained by its conversion either to galactocerebrosides or to fatty aldehydes, mainly by the action of LOC100155321 (ACER2), LOC100738292 (SPHK2) and SGPL1 gene products. Building around the aforesaid speculations and the literature evidences from model organisms, we propose sphingolipids such as ceramide, sphingosine and sphigosine-1-phosphate and genes involved in sphingolipid metabolic pathway such as GALC, LOC100152988 (KDSR), SGMS1, SGMS2, SMPD1 and SMPD4 as candidate biomarkers to be investigated for their involvement in porcine steroid hormone biosynthesis and androstenone biosynthesis pathways. From Figure 5 it can be seen that several other interactions positive in either one of the phenotypes, but we were unable to find literature or database evidences to explain and support these interactions.

### Fatty Acid Metabolism

**Figure 6:**
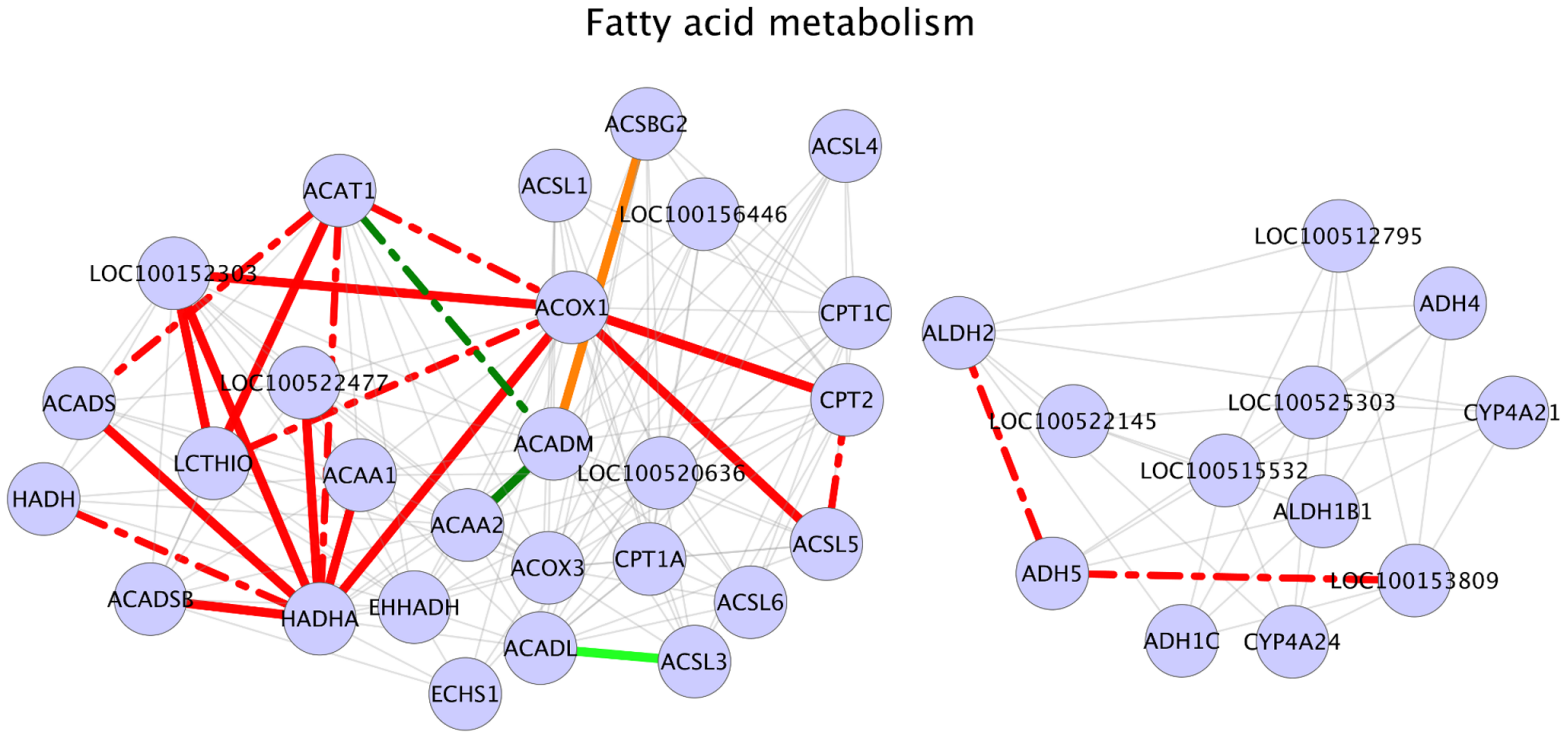
Fatty acid metabolism. Legend: nodes – genes, edges – interactions with significant z-scores. Edge legend: Grey edges: Non significant interactions, part of KEGG network data. Red solid edges: interactions positive and significant in HA samples, negative in LA samples. Red dashed edges: interactions positive and significant in HA samples, positive in LA samples. Orange solid edges: interactions positive in HA samples, negative and significant in LA samples. Orange dashed edges: interactions negative in HA samples, negative and significant in LA samples. Dark green solid edges: interactions positive and significant in LA samples, negative in HA samples. Dark green dashed edges: interactions positive and significant in LA samples, positive in HA samples. Light green solid edges: interactions positive in LA samples, negative and significant in HA samples. Light green dashed edges: interactions negative in LA samples, negative and significant in HA samples.

Fatty acid metabolism was also one of the enriched pathways in our analysis results (Table 3). The beta oxidation (catabolic) part of fatty acid metabolism breaks down fatty acids to acetyl-CoA which then enters TCA cycle and electron transport chain metabolic pathways for energy generation. We found that in our results, a total of 23 interactions in fatty acid metabolism were significant (Figure 6). Out of the 23 interactions, 20 interactions were positive in HA samples and 3 interactions were positive in LA samples (Figure 6), possibly indicating an active fatty acid metabolic pathway in HA animals. Eight out of the twenty interactions in HA samples had the gene HADHA as one of the interaction partners (Figure 6). The gene HADHA codes for mitochondrial trifunctional protein alpha subunit, an enzyme necessary for the final steps mitochondrial beta oxidation of fatty acids [75]. This suggests that the fatty acid oxidation might be highly active in HA samples, oxidizing fatty acids to acetyl-CoA. Acetyl-CoA is also the starting molecule for de novo synthesis of cholesterol. Our results also show that the interactions between acetyl-CoA acetyltransferase genes and HADHA were also positive in HA animals. These interactions were: ACAT1– HADHA interaction and LOC100152303 (ACAT2) – HADHA interaction (Figure 6). Enzymes encoded by the genes ACAT1 and LOC100152303 (ACAT2) belong to the thiolase family of enzymes and the major function of these enzymes is catalyzing the synthesis of acetoacetyl-CoA from two units of acetyl-CoA [76]. Acetoacetyl-CoA generated as a result of this reaction enters mevalonate pathway leading to cholesterol synthesis [77]. It has been shown that cholesterol used in steroidogenesis could be derived from cholesteryl ester mobilization, selective uptake of cholesteryl esters or de novo synthesis of cholesterol in cytosol [46]. In this regard, we hypothesize that acetoacetyl-CoA derived from an active fatty acid metabolic pathway in HA animals could have enhanced the de novo synthesis of cholesterol in testis tissues of HA animals. Cholesterol synthesized in this manner might be also entering steroidogenic and androstenone biosynthetic pathways finally resulting in higher amounts of androgens in these animals.

### Cyclic AMP – PKA/PKC Signaling

**Figure 7:**
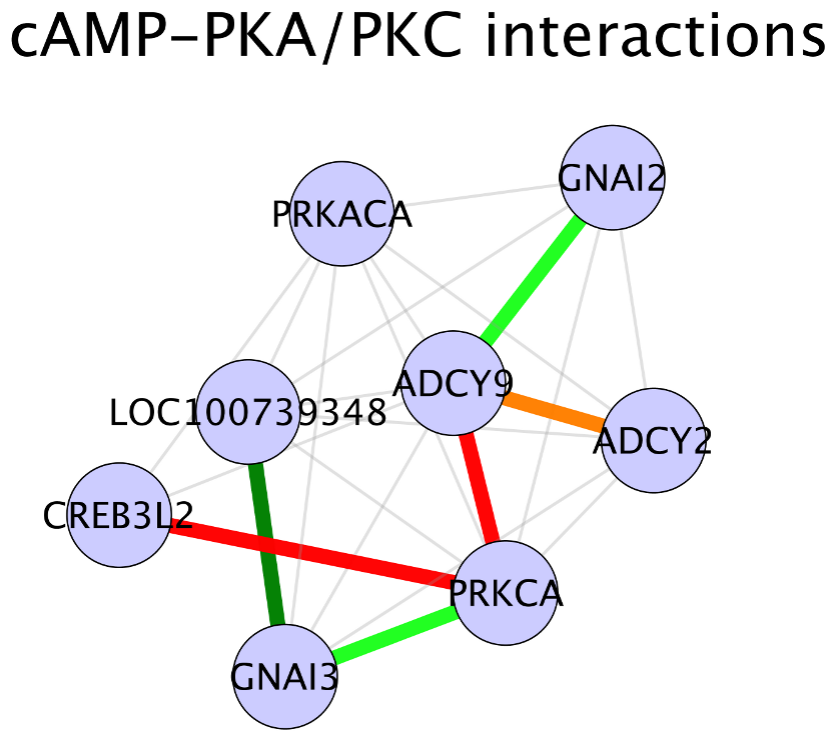
cAMP/PKA related interactions. Legend: nodes – genes, edges – interactions with significant z-scores. Edge legend: Grey edges: Non significant interactions, part of KEGG network data. Red solid edges: interactions positive and significant in HA samples, negative in LA samples. Red dashed edges: interactions positive and significant in HA samples, positive in LA samples. Orange solid edges: interactions positive in HA samples, negative and significant in LA samples. Orange dashed edges: interactions negative in HA samples, negative and significant in LA samples. Dark green solid edges: interactions positive and significant in LA samples, negative in HA samples. Dark green dashed edges: interactions positive and significant in LA samples, positive in HA samples. Light green solid edges: interactions positive in LA samples, negative and significant in HA samples. Light green dashed edges: interactions negative in LA samples, negative and significant in HA samples.

In addition to the interactions in significant pathways, we also found additional interactions which could be relevant in maintaining steroidogenesis in porcine testes tissues. A number of these identified interactions were part of cAMP (cyclic AMP)/PKA signaling, although this pathway was neither represented in the KEGG pathway interaction data that we used in this analysis nor enriched for significant interactions. Cyclic AMP/PKA signaling pathway is one of the primary signaling cascades maintaining and regulating steroidogenesis [78]. Cyclic-AMP/PKA signaling pathway activation of steroidogenesis is initiated by trophic hormones, which activate G-proteins. G-proteins stimulate adenylate cyclases, thus increasing the levels of intracellular cAMP which further activates protein kinase A (PRKACA). An activated protein kinase A phosphorylates transcription factors such as steroidogenic factor 1 (NR5A1), GATA binding protein 4 (GATA4), cAMP response-element binding protein (CREB) and cAMP response element modulator (CREM) which activate the genes involved in steroidogenesis [78]. We found that the interaction between the genes ADCY9 and PRKCA was significant and positive in HA samples (Figure 7). The gene ADCY9 codes for the enzyme adenylate cyclase type 9, which catalyzes the conversion of ATP to cyclic AMP and diphosphate [79]. PRKACA, as mentioned above, upon cAMP activation phosphorylates certain transcription factors which activates the genes involved in steroidogenesis. The interaction between the genes PRKCA and CREB3L2 was also found to be significant and positive in HA animals. CERB3L2 is described as cAMP responsive element binding protein (CREB) 3-like 2, but whether the transcription factor encoded by this gene activates the genes involved in steroidogenesis is unknown as of now. Interestingly, we also found that two interactions involving adenylate cylases class of genes and guanine nucleotide binding protein class of genes were positive in LA animals. These interactions were: ADCY9 - GNAI2 interaction and LOC100739348 (ADCY8) - GNAI3 interaction (Figure 7). Contrary to the interactions observed in HA animals, the interactions found in LA animals were inhibitory. One of the functions of guanine nucleotide binding protein family is the inhibition of adenylate cylases [80], indicating that GNAI gene products were possibly inhibiting the action of ADCY gene products in LA animals. Another LA positive interaction in our results was the interaction between the genes ADCY2 and PRKCA. ADCY2, similar to other adenylate cyclases, catalyzes the synthesis of cAMP. Gene PRKCA codes for the alpha subunit of the protein protein kinase C (PKC). In a similar manner to PKA, PKC has also been shown to be activated by trophic hormones and stimulates adenylate cyclase activity indicating that in addition to PKA, PKC also influences gonadal steroidogenesis [78], [81]. But studies done over the years have demonstrated that PRKCA (PKC) is a weak inducer of steroidogenesis and that progesterone synthesis in rat Leydig cells is only moderately elevated by PKC activation [82]–[84]. In contrast, Fleury et al. [85] showed that mutation of PRKACA (PKA) phosphorylation sites in StAR protein reduced steroidogenesis by 70–80%. These published evidences points out PRKACA (PKA) as a major steroidogenesis activator and PRKCA (PKC) as an auxiliary activator of steroidogenesis. By piecing together our interaction results at the genomic level and information from published articles, we speculate that in HA animals an active cAMP/PKA signaling results in higher steroidogenic activity. But in case of LA animals, although cAMP/PKC based signaling of steroidogenesis was active, the inhibition of adenylate cylases by guanine nucleotide binding proteins might be slowing down the steroid hormone synthesis machinery and thus could be affecting androstenone synthesis.

**Figure 8:**
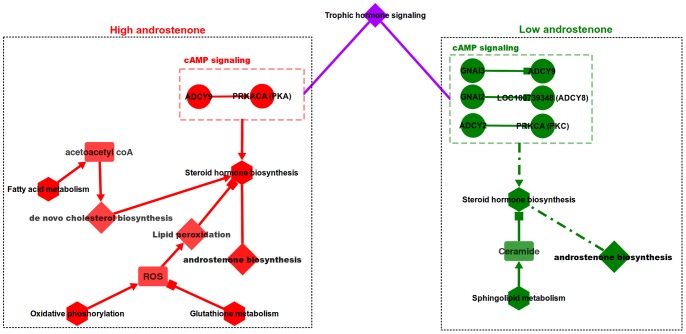
Hypothetical network visualizing the proposed difference maintaining and regulating steroidogenesis and androstenone biosynthesis in high and low androstenone boars. Legend: Circular nodes: genes, hexagonal nodes: enriched pathways, diamond nodes: pathways that might be involved in steroidogenesis, but not found in results, rectangular nodes: metabolites from pathways. In high androstenone samples, steroidogenesis was activated by cAMP-PKA signaling, lipid peroxidation activity of ROS was inhibited by metabolites from glutathione metabolism pathway and de novo synthesis of cholesterol as a result of an active fatty acid metabolism activity might have boosted steroidogenesis and androstenone synthesis. In low androstenone samples, weak cAMP-PKC signaling of steroidogenesis and inhibition of steroidogenesis by ceramides synthesized from sphnigolipid metabolim pathway might have lead to weak steroidogenesis and hence, low androstenone synthesis.

**Figure 9:**
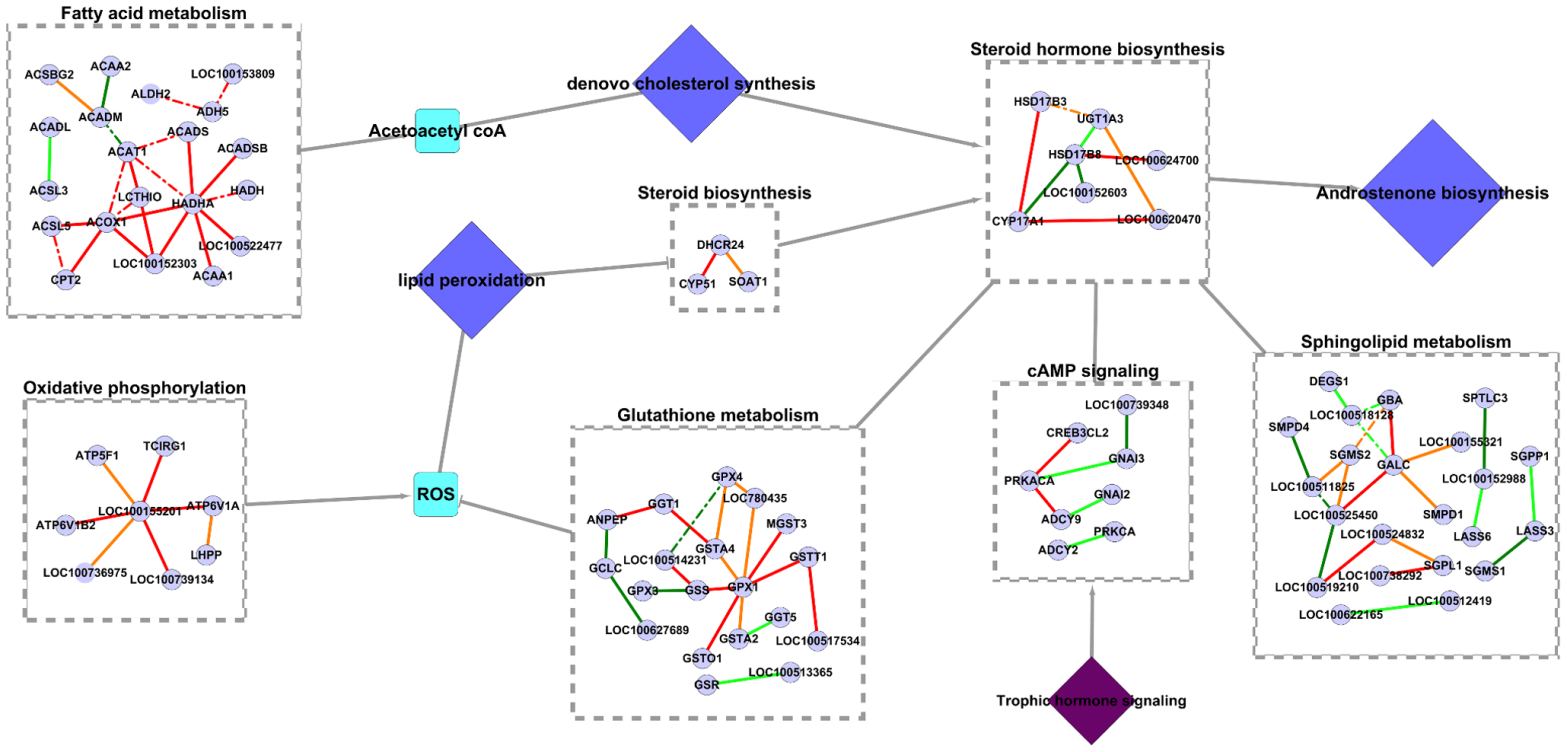
Hypothetical network showing the metabolic pathways affecting steroidogenesis and androstenone biosynthesis. Legend: Interactions inside each pathway shows the significant interactions from analysis with genes as nodes and significant KEGG pathway interactions as edges. Blue diamond nodes: pathways that might be involved in steroidogenesis, but not found in results. Cyan colored nodes: chemical compound or molecules synthesized in pathways. Purple node: external stimulus in the form of hormone signaling. Grey solid edges: hypothetical interactions based on information from literature. Very light blue circular nodes: genes involved in significant interactions. Red dashed edges: interactions positive and significant in HA samples, positive in LA samples. Orange solid edges: interactions positive in HA samples, negative and significant in LA samples. Orange dashed edges: interactions negative in HA samples, negative and significant in LA samples. Dark green solid edges: interactions positive and significant in LA samples, negative in HA samples. Dark green dashed edges: interactions positive and significant in LA samples, positive in HA samples. Light green solid edges: interactions positive in LA samples, negative and significant in HA samples. Light green dashed edges: interactions negative in LA samples, negative and significant in HA samples. In HA animals, steroid synthesis and androstenone synthesis might be activated by cAMP-PKC signaling and further boosted by de novo cholesterol synthesis by virtue of an active fatty acid metabolism pathway and anti lipid peroxidation activity of members of glutathione metabolism pathway. In LA animals steroid hormone synthesis and androstenone synthesis could have been affected by weak cAMP-PKA signaling and inhibition of steroidogenesis by ceramides synthesized from sphingolipid metabolic pathway.

Amalgamating the speculations discussed above, we hypothesize that the combined action of cAMP-PKA/PKC signaling, glutathione metabolism, sphingolipid metabolism and fatty acid metabolism was affecting steroid hormone synthesis and therefore androstenone biosynthesis in both HA and LA animals. In HA samples, one of the factors contributing to high androstenone could be that steroidogenesis and hence androstenone synthesis in these animals were activated by trophic hormone signaling through cAMP-PKA (PRKACA) signaling. Additionally, these pathways could have been further boosted by anti lipid peroxidation activity by members of glutathione metabolism pathway and de novo synthesis of cholesterol as a result of an active fatty acid metabolic pathway (Figure 8). In case of LA samples, it could be assumed that a weak cAMP-PKC (PRKCA) based signaling of steroidogenesis activation and synthesis of ceramide by sphingolipid metabolic pathway, which inhibits steroidogenesis could be the reason for a low steroidogenesis and hence low androsteonone synthesis (Figure 8). Figure 8 shows a hypothetical metabolic pathway illustrating the proposed hypothesis of steroidogenesis and androstenone synthesis regulation in low and high androstenone phenotypes. Network diagram in Figure 8 was generated using Omix [86]. Figure 9 is an illustration of the proposed hypothesis at an interaction level, showing significant interactions in the mentioned pathways. Figure S2 shows steroid hormone biosynthesis, androstenone biosynthesis pathway and associated signaling and metabolic pathways which either affect sterodiogenesis and androstenone biosynthesis or were affected by androgens. The purpose of this diagram is to visualize the interactions which affect steroid hormone synthesis along with the pathways that are affected by the androgens. At this point, it should be taken into consideration that the speculations and assumptions presented here are based on in-silico evidences from experiments done at the genomic level and needs to be validated.

## Conclusion

In this study, we aimed to identify and study the key pathways and interactions in porcine steroidogenesis and androstenone biosynthesis using an integrative approach based analysis method. In the light of the results from our analysis, we hypothesize that pathways such as glutathione metabolism, sphingolipid metabolism, fatty acid metabolism and cAMP-PKA/PKC signaling were fundamental in maintaining and regulating steroidogenesis and hence androstenone biosynthesis in both high and low androstenone animals. We theorize that in high androstenone animals, steroidogenesis was activated by cAMP-PKA signaling and that the anti lipid peroxidation activity of glutathione metabolism and de novo synthesis of cholesterol as a result of an active fatty acid metabolism activity might have boosted steroidogenesis and androstenone metabolism. In low androstenone animals, we postulate that a weak cAMP-PKC activation of steroidogenesis and regulatory action of ceramides on steroidogenesis might have contributed to a weak steroid hormone synthesis and hence, low levels of androstenone synthesis. To conclude, the combined effect of these key differences in the metabolic and signaling pathways of high and low androstenone animals could be the determining factor for the levels of steroidogenesis and androstenone biosynthesis in these animals. The major objective of this study was hypothesis generation on porcine androstenone biosynthesis based on existing data resources and the results and hypotheses presented in this work are based on evidences at the genomic level from an in-silico study. To the best of our knowledge, this work is the first attempt at modeling the biochemical machinery behind divergent androstenone biosynthesis via a hypothesis driven approach. In this work, the biochemical network modeling approach was chosen for aggregating a priori knowledge and to support model driven data analysis and validation for porcine androstenone research community. The collective intelligence of the androstenone research community is crucial to challenge (test) the proposed hypotheses using currently existing or newly generated data and to validate the regulatory mechanisms proposed in this analysis. In order to confirm and validate the findings from this work, additional wet laboratory experiments at the genome, proteome or metabolome level are necessary.

## Supporting Information

Figure S1 **Schematic diagram of entire workflow adapted in this analysis.** Legend: White parallelograms with grey outline: Input/output data and results. White cylinders with red outline: data from external databases. Rectangles with light blue shades: various tools and analysis processes used in this workflow.
(TIFF)

Figure S2 **Hypothetical interaction network.** Hypothetical network at pathway level showing the metabolic pathways affecting steroidogenesis and androstenone biosynthesis and pathways that are affected by steroid hormones. Legend: Grey hexagonal nodes: pathways that were enriched for significant interactions. Blue diamond nodes: pathways that might be involved in steroidogenesis, but not found in results. Purple diamond node: external stimulus in the form of hormone signaling. Cyan rectangular nodes: chemical compound or molecules synthesized in pathways. Dark blue solid edges: Interactions between enriched pathways (source: KEGG database). Dark blue solid double line edges: Edge between a compound and a pathway showing a compound synthesized in pathway. Light blue dashed edges: hypothetical interactions based on information from literature.
(TIFF)

File S1 **Significant interaction network.** Cytoscape (.xgmml) file containing the significant interactions visualized as a network along with additional information such as LA and HA correlation coefficients, raw read counts for each gene, empirical p-value and correlation type for each interaction, generated using Cytoscape version 2.8.2. To visualize the network, please download and install Cytoscape (http://www.cytoscape.org/last accessed November 4, 2013) and import the .xgmml file by: File → Import → Network (Multiple file types). Additional information on importing files is given in (http://wiki.cytoscape.org/GettingStarted last accessed November 4, 2013).
(XGMML)

File S2 **Enriched network.** Cytoscape (.xgmml) file containing network visualization of significant interactions in enriched pathways and each edge in this network holds attributes containing KEGG pathway identifiers and names of enriched pathways, generated using Cytoscape version 2.8.2.
(XGMML)
