# Supplementary figures and images for: Pathway Based Analysis of Genes and Interactions Influencing Porcine Testis Samples from Boars with Divergent Androstenone Content in Back Fat
Source: PLoS One. 2014 Mar 10;9(3):e91077. doi: 10.1371/journal.pone.0091077 (PMC3948775; doi:10.1371/journal.pone.0091077)

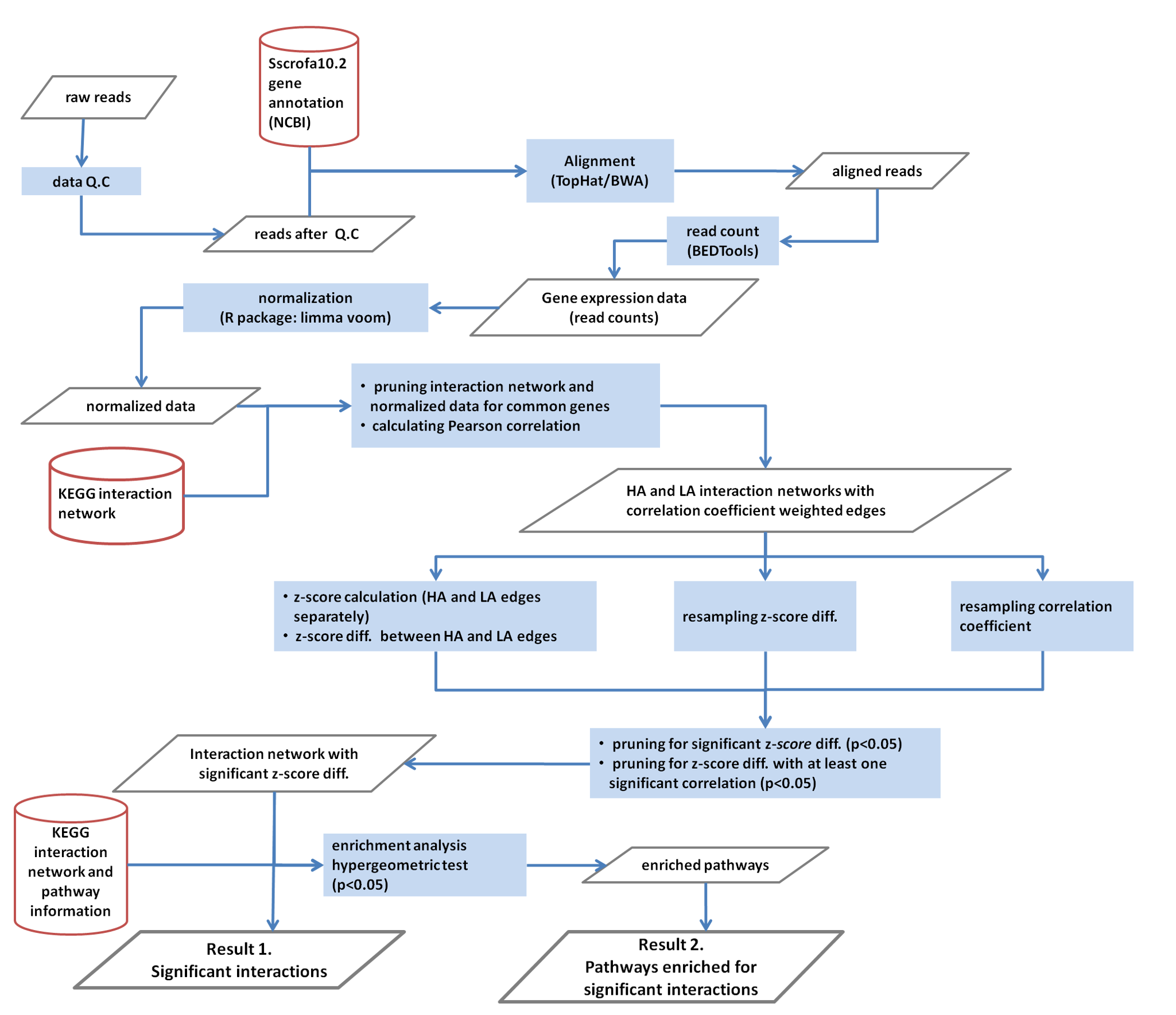

Supplement: Figure S1 — Schematic diagram of entire workflow adapted in this analysis. Legend: White parallelograms with grey outline: Input/output data and results. White cylinders with red outline: data from external databases. Rectangles with light blue shades: various tools and analysis processes used in this workflow. (TIFF) [file pone.0091077.s001.tiff]

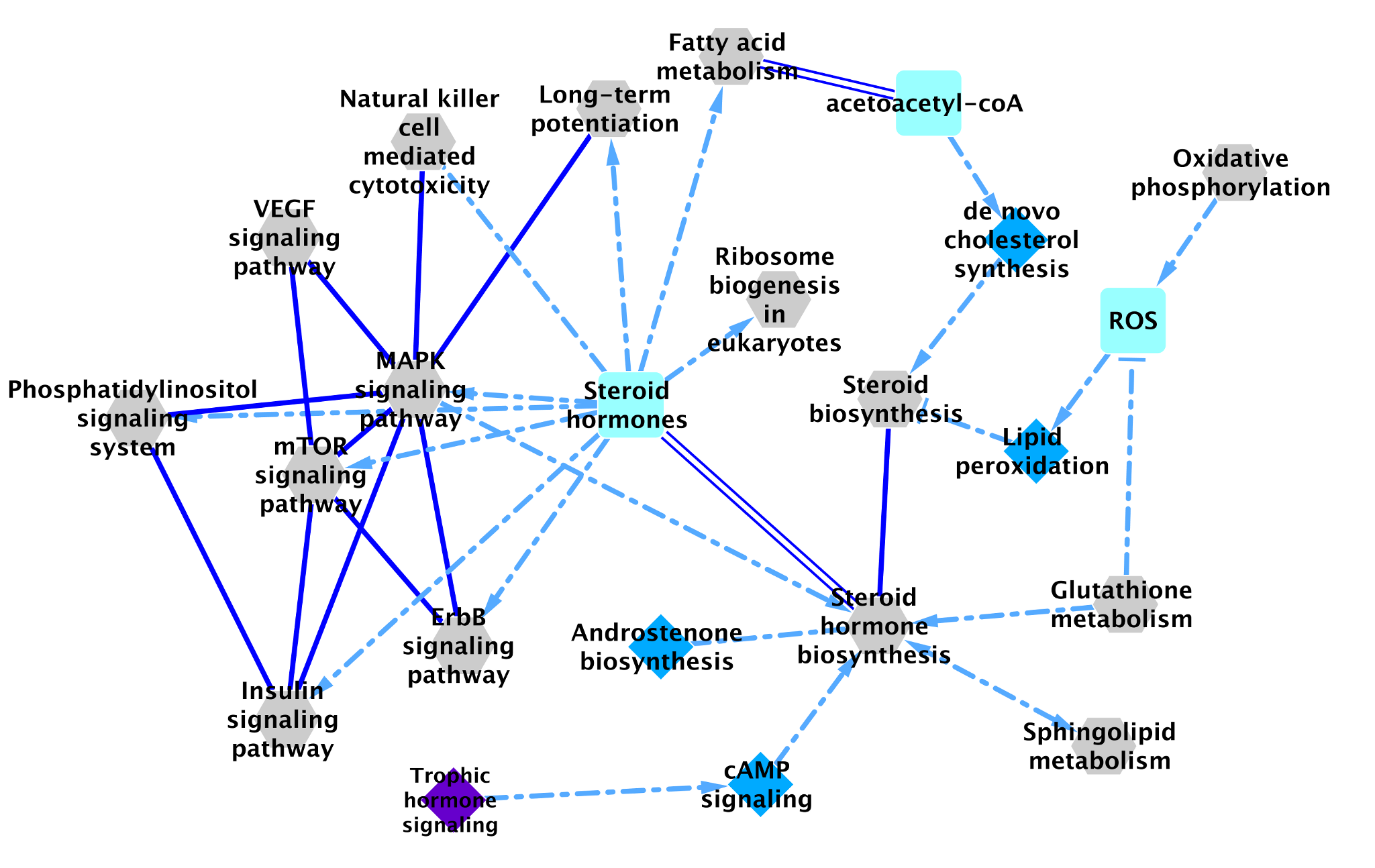

Supplement: Figure S2 — Hypothetical interaction network. Hypothetical network at pathway level showing the metabolic pathways affecting steroidogenesis and androstenone biosynthesis and pathways that are affected by steroid hormones. Legend: Grey hexagonal nodes: pathways that were enriched for significant interactions. Blue diamond nodes: pathways that might be involved in steroidogenesis, but not found in results. Purple diamond node: external stimulus in the form of hormone signaling. Cyan rectangular nodes: chemical compound or molecules synthesized in pathways. Dark blue solid edges: Interactions between enriched pathways (source: KEGG database). Dark blue solid double line edges: Edge between a compound and a pathway showing a compound synthesized in pathway. Light blue dashed edges: hypothetical interactions based on information from literature. (TIFF) [file pone.0091077.s002.tiff]
